# Targeting mitochondrial dysfunction with small molecules in intervertebral disc aging and degeneration

**DOI:** 10.1007/s11357-021-00341-1

**Published:** 2021-02-26

**Authors:** Morteza Saberi, Xiaolei Zhang, Ali Mobasheri

**Affiliations:** 1 Department of Life Science Engineering, Faculty of New Sciences and Technologies, University of Tehran, Tehran, Iran; 2 Department of Orthopaedics, The Second Affiliated Hospital and Yuying Children’s Hospital of Wenzhou Medical University, Wenzhou, Zhejiang Province China; 3 Key Laboratory of Orthopaedics of Zhejiang Province, Wenzhou, Zhejiang Province China; 4 Research Unit of Medical Imaging, Physics and Technology, Faculty of Medicine, University of Oulu, Oulu, Finland; 5 Department of Regenerative Medicine, State Research Institute Centre for Innovative Medicine, Vilnius, Lithuania; 6 Departments of Orthopedics, Rheumatology and Clinical Immunology, University Medical Center Utrecht, Utrecht, The Netherlands; 7 Department of Joint Surgery, First Affiliated Hospital of Sun Yat-sen University, Guangzhou, Guangdong China

**Keywords:** Intervertebral disc (IVD), Degeneration, Mitochondrial dysfunction, Small molecule, Therapeutic, Mitophagy, Growth factor

## Abstract

The prevalence of rheumatic and musculoskeletal diseases (RMDs) including osteoarthritis (OA) and low back pain (LBP) in aging societies present significant cost burdens to health and social care systems. Intervertebral disc (IVD) degeneration, which is characterized by disc dehydration, anatomical alterations, and extensive changes in extracellular matrix (ECM) composition, is an important contributor to LBP. IVD cell homeostasis can be disrupted by mitochondrial dysfunction. Mitochondria are the main source of energy supply in IVD cells and a major contributor to the production of reactive oxygen species (ROS). Therefore, mitochondria represent a double-edged sword in IVD cells. Mitochondrial dysfunction results in oxidative stress, cell death, and premature cell senescence, which are all implicated in IVD degeneration. Considering the importance of optimal mitochondrial function for the preservation of IVD cell homeostasis, extensive studies have been done in recent years to evaluate the efficacy of small molecules targeting mitochondrial dysfunction. In this article, we review the pathogenesis of mitochondrial dysfunction, aiming to highlight the role of small molecules and a selected number of biological growth factors that regulate mitochondrial function and maintain IVD cell homeostasis. Furthermore, molecules that target mitochondria and their mechanisms of action and potential for IVD regeneration are identified. Finally, we discuss mitophagy as a key mediator of many cellular events and the small molecules regulating its function.

## Background

Low back pain (LBP) is one of the leading causes of disability worldwide and IVD degeneration is postulated to be the main pathogenic process involved [1, 2]. In a healthy IVD, the balance between anabolic and catabolic processes maintains ECM homeostasis; however, aging and persistent mechanical stress can disrupt IVD metabolism forcing an imbalance between the expression of catabolic factors (such as pro-inflammatory cytokines and matrix metalloproteinases), and anabolic mediators (such as growth factors), eventually leading to the loss of ECM homeostasis, destruction of macromolecules, and the subsequent development of IVD degeneration [3–5]. The pathophysiology of IVD degeneration is characterized by the loss of ECM components including collagen fibers and proteoglycans as well as the loss of cellularity [6].

Mitochondria perform crucial functions including energy production and intracellular calcium ion Ca^2+^ (homeostasis, regulation of apoptosis, and innate immunity). However, their primary function is related to energy production. They are the cell’s main powerhouse, providing metabolic energy in the form of adenosine triphosphate (ATP) molecules [7, 8]. Because of hypoxic conditions in the microenvironment of the IVD, ATP production mainly relies on glycolysis [9, 10]. Hypoxia-inducible factor 1-α (HIF-1α) is upregulated under hypoxia and mediates glycolysis-oxidative phosphorylation (OXPHOS) interplay to regulate metabolism [9]. Mechanical overload alters mitochondrial membrane potential (∆*Ψ*_*m*_) in IVD cells. Mitochondria with disrupted membrane potential further mediate cell proliferation, induce apoptosis, and lead to IVD degeneration [11, 12]

Mitochondria are also the main producers of highly reactive radical species specifically reactive oxygen species (ROS) in cells. Accumulation of ROS alters the cellular metabolism, ATP production, and the synthesis of both nuclear and mitochondrial DNA encoded genes [13]. Accumulation of excessive ROS also leads to oxidative stress resulting in mitochondrial dysfunction [14]. Depolarized mitochondria are characterized by the loss of ∆*Ψ*_*m*_ that precedes intrinsic apoptosis [15]. Dysfunctional mitochondria fail to supply cellular energy homeostasis, which is an incident in many age-related and inflammatory joint diseases. Depolarized mitochondria are also susceptible to Ca^2+^ overload which contributes to programmed cell death such as apoptosis [16]. Reversing mitochondrial dysfunction can potentially counteract the oxidative stress and consequent cell death that can accelerate IVD degeneration [17]. Hence, the regulation of mitochondrial function is an emerging approach to delay IVD degeneration.

Mitochondria-driven ROS are mainly produced at I_f_ and I_Q_ sites of complex I during electron transport to coenzyme Q (CoQ) and in a lower degree at Q_I_ site of complex III in the presence of ubisemiquinone [18, 19]. Electron transfer chain (ETC) is not always 100% efficient and up to 4% electron leakage happens before reaching the final electron acceptor in cytochrome c oxidase (complex IV) [20]. Superoxide $\left({\mathrm{O}}_2^{-.}\right)$ as the proximal mitochondrial ROS is produced when the leaked electron is caught by an oxygen molecule. Superoxide is a highly reactive molecule with a short lifespan. It is dismutated to the more stable molecule, hydrogen peroxide (H_2_O_2_). Superoxide dismutase (SOD) enzyme isoforms, SOD1, SOD2, and SOD3 convert superoxide to hydrogen peroxide in cytosol, mitochondria, and ECM, respectively [21]. Hydrogen peroxide can later be decomposed to water by enzymes with anti-oxidant activity or turns to hydroxyl radical (OH^°^) and hydroxide ion (OH^−^) via the Fenton reaction [22]. Hydroxyl radical is found as the main ROS in cultured IVD cells [22]. Moreover, ${\mathrm{O}}_2^{-.}$ can react with nitroxide radical (NO^°^) and form the highly potent oxidant, peroxynitrite (ONOO^−^). Peroxynitrite is more potent than NO^°^ as a degenerative signal in chondrocytes and progression of IVD degeneration [23, 24].

None of the clinically available LBP treatments including conservative treatments and surgical interventions addresses the regeneration of degenerated IVD though they may manage the pain and alleviate the symptoms [25–27]. Molecular therapy as a branch of regenerative medicine has been utilized to address the available treatment shortcomings [28]. The efficacy of various growth factor-based therapeutics as well as biopolymers for the treatment of degenerate and age-associated disc diseases has been extensively investigated. For instance, calcitonin, which is a thyroid gland-secreted polypeptide hormone, induces more vitamin D production and inhibits bone resorption [29, 30]. Calcitonin is thought to suppress IVD degeneration through maintaining matrix homeostasis in the IVD and inhibiting bone loss in the adjacent vertebral body [31–33]. Growth factors such as transforming growth factor-β1 (TGF-β1), bone morphogenetic proteins (BMPs), and basic fibroblast growth factor (bFGF) are other examples of proteins with pro-regenerative [34]. TGF-β1 can reverse mitochondrial dysfunction and abate oxidative stress in AF cells and decrease mitochondria-mediated apoptosis [35]. Insulin and insulin-like growth factor-1 (IGF-1) have been demonstrated to induce chondrogenic differentiation and reduce the progression of IVD degeneration in vitro and in vivo, and also in animal models of type 2 diabetes mellitus [36–39]. Growth factors possess therapeutic properties. However, there are a number of important shortcomings associated with their use and extensive discussion of the role of growth factors and small peptides is beyond the scope of this review article. Therefore, this review focuses on the role of small molecules as novel therapeutic agents for IVD degeneration.

Using small molecules may be a promising approach to potentially regulate intracellular signaling pathways and target mechanisms underlying common diseases [40]. Thus far, inflammation-targeting molecules and ROS scavenging therapeutic agents have been reviewed in the field of IVD degeneration [41, 42], aiming to provide a broader perspective of basic studies that have been done to evaluate the efficacy of small molecules regulating mitochondrial function and their underlying mechanisms to control IVD degeneration progression both in vitro and in vivo. Finally, the role of mitochondrial autophagy (mitophagy) in mitochondrial dysfunction and its regulatory molecules are discussed.

## Mitochondrial dysfunction in IVD degeneration

### Interplay between mitochondrial dysfunction-oxidative stress

**Fig. 1:**
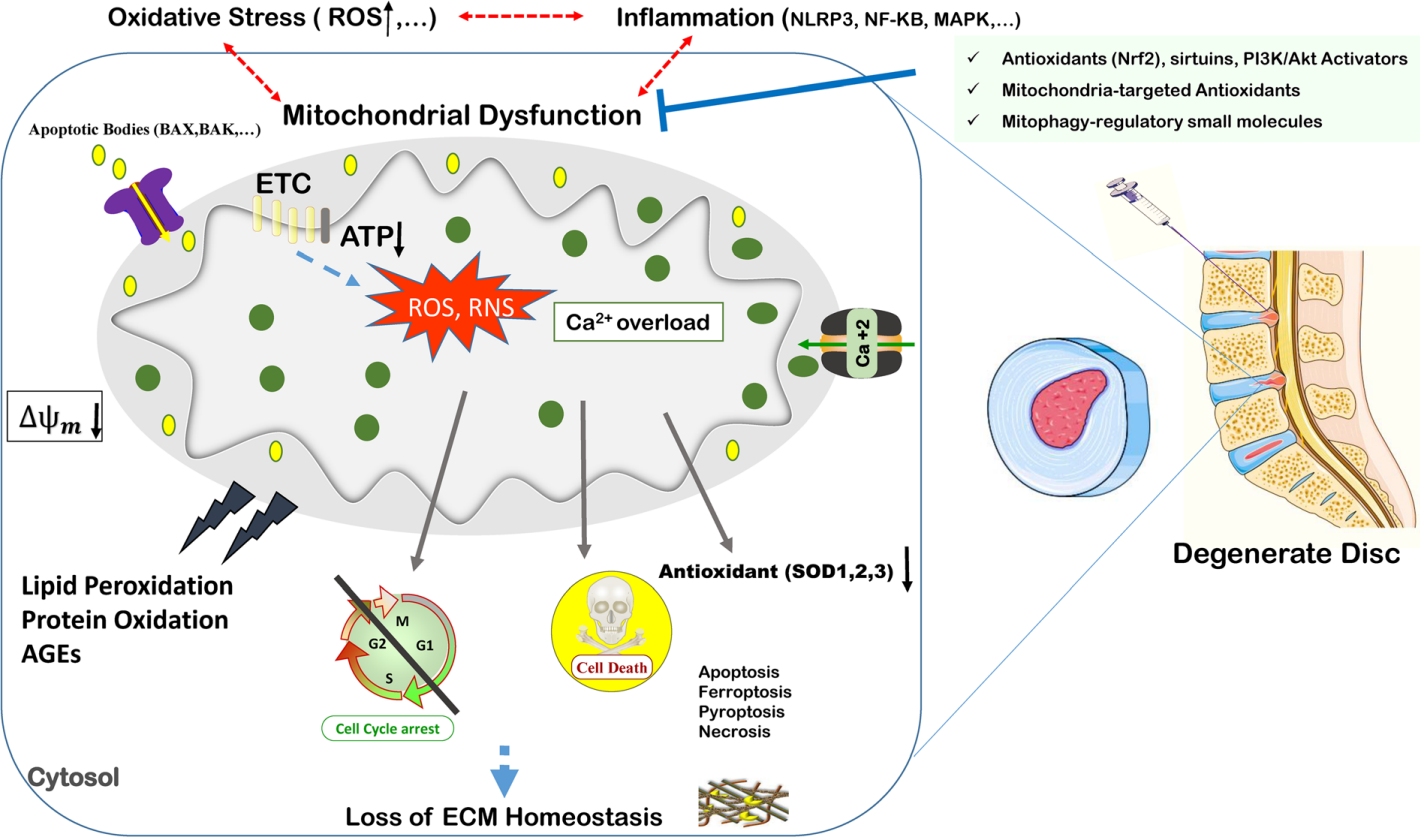
Consequences of intervertebral disc degeneration. The interplay between mitochondrial dysfunction, oxidative stress, and inflammation

In addition to their pivotal role as energy providers, mitochondria are the main source of ROS generation in cells [43]. ROS including free radicals and non-radical molecules as well as reactive nitrogen species (RNS) are produced as byproducts of electron transfer in the mitochondrial respiratory chain (MRC) [18, 19]. Physiological levels of ROS and RNS are beneficial through mediating cellular redox signaling and homeostasis [44, 45]. Nonetheless, excessive production of ROS and RNS or impaired anti-oxidant defense mechanism induces oxidative stress. Oxidative stress can depolarize the mitochondrial membrane, leading to electron leakage, the release of apoptotic molecules, cellular *Ca*^2+^ overload, and cellular metabolism balance perturbation through ATP production imbalance [46–48]. Conversely, dysfunctional mitochondria with dissipated membrane potential exhibit exacerbated cellular stress and mediate multiple adverse cellular events including lipid peroxidation and protein oxidation, cell senescence, and cell death [49], indicating the interplay between mitochondrial dysfunction and induction of oxidative stress (Fig. 1). The level of malondialdehyde (MDA) and advanced oxidation protein products (AOPP) as lipid peroxidation and protein oxidation biomarkers has been demonstrated to be enhanced with age in IVD tissue of rats, while the level of SOD decreases [50]. Advanced glycation end products (AGEs) are a type of sugar-modified proteins, lipids, and nucleic acids, which are found in IVD. Among the identified AGEs, carboxymethyl lysine (CML), pentosidine, and methylglyoxal have been found in degenerate IVD [51–53]. Song et al. reported that AGE-induced oxidative stress targeted the mitochondrial sirtuin, sirtuin 3, impaired its function, and led to mitochondria-mediated apoptosis in human-derived NP cells. Mitochondrial redox homeostasis preservation via restoring sirtuin 3 function, nevertheless, inhibited apoptosis and alleviated IVD degeneration [54].

Senescence is another pathophysiological consequence of oxidative stress and is implicated in mitochondrial dysfunction. Senescent cells are non-proliferative, apoptosis-resistant cells with dysregulated metabolic activity, arisen from persistent stress and cellular damage [55]. Many factors including telomere shortening, endogenous- and exogenous-driven stressors, and mitochondrial dysfunction are implicated in the cell entrance to irreversible growth arrest state or the so-called senescence [55]. The telomere-based P53-P21-PRB and stress-induced P16-PRB are the central pathways regulating cell senescence [55]. Aging correlates with mitochondrial dysfunction and accumulation of senescent cells. Nucleus pulposus (NP) samples from patients with degenerate IVD demonstrate more senescent cells with higher P53 and P21 expression and SA-β-gal activity, lower telomere length from old cases relative to younger patients [56]. Mitochondria directly control the P16-RPB pathway of senescence in a P38/MAPK (mitogen-activated protein kinase)-dependent manner and indirectly phosphorylate and activate P53 by inducing DNA damage response as well [57]. Given the significance of mitochondrial dysfunction in cell senescence, preserving ∆*Ψ*_*m*_ is an approach to protect mitochondrial function and decelerate cell senescence. ∆*Ψ*_*m*_ preservation inhibits excessive mitophagy and the loss of mitochondria [58]. Mitochondrial dysfunction and cellular senescence are implicated in the process of IVD aging [59]. ∆*Ψ*_*m*_ declination with age and proton leakage enhancement has been observed in IVD cells [47]. ATP production declines with aging in NP and annulus fibrosis (AF) cells [47]. Hartman et al. showed that mitochondria undergo morphological changes with aging as the number of mitochondria reduces while mean volume per mitochondria is enhanced in NP cells [47]. In contrast, enhancement of ATP synthase activity, ATP production, and mitochondrial numbers were reported in senescent NP cells [60]. Taken together, there is an interplay between mitochondrial dysfunction, aging, and cell senescence in the onset and progression of IVD degeneration; however, it is presently unclear as to which process comes first.

### Mitochondrial dysfunction-inflammation cross talk—the role of NLRP3 inflammasome

IVD degeneration is characterized by high ROS content, impaired anti-oxidant activity, and inflammatory responses. Oxidative stress, especially driven from mitochondrial dysfunction, and inflammation are mechanistically linked and eventually lead to IVD degeneration [46, 49, 61] (Fig. 1). The inflammasome consists of a multiprotein complex which recognizes exogenous pathogenic factors including microorganisms, toxins, lipopolysaccharide (LPS), radiation, oxidative stress as well as endogenous danger signals, and mediates innate immune response [62, 63]. Nucleotide-binding oligomerization domain, leucine-rich repeat, and pyrin domain-containing protein (NLRPs) inflammasomes are a distinct sub-type of NOD-like receptors (NLRs) inflammasome, a cytoplasmic set of pattern-recognition receptors (PRRs), which recognize pathogen-associated molecular patterns/damage-associated molecular patterns (PAMPs/DAMPs) through toll-like receptors (TLRs) and stimulate immune response [62, 64, 65]. NLRPs comprise of fourteen isoforms, NLRP 1-14, in which the NLRP3 inflammasome is the most well-known NLRP [62]. NLRP3 inflammasome consists of an NLRP3 scaffold, a cysteine protease and proinflammatory caspase, procaspase 1, and apoptosis-associated speck-like protein, containing a caspase-recruitment domain (ASC) adaptor. NLRP3-ASC interaction and subsequent activation of procaspase 1 mediate activation and maturation of proinflammatory cytokines such as pro-IL-1β and pro-IL-18 which induce inflammatory response including NF-κB activation and lead to a type of cell death called “pyroptosis” [62, 63, 66]. Activation of the NLRP3 inflammasome requires two critical steps: priming and activation. Upon inflammatory, toxic, or microbial stimulus, NLRP3 priming occurs. Deubiquitylated NLRP3 and caspase1 make an association with mitochondria in a ROS-dependent manner and they independently bind to the externalized cardiolipin in the outer mitochondrial membrane (OMM). ASC recruitment to mitochondria and activation of NLRP3 inflammasome complex occurs in a *Ca*^2+^-dependent way [67, 68]. In addition to the pro-inflammatory signal, mtROS has been reported to be needed for NLRP3 priming [69]. Several mechanisms have been introduced for NLRP3 activation. NLRP3 may be activated in a ROS-dependent way by ROS producers such as ATP and silica and/or in a ROS-independent way by NLRP3 agonist, linezolid [70]. Thioredoxin-interacting protein (TXNIP) is the inhibitor of thioredoxin, a cytoprotective oxidoreductase with ROS scavenging activity. TXNIP expression leads to oxidative stress and is implicated in inflammation and mitochondrial damage [71]. NLRP3 binding of TXNIP led to inflammatory response, oxidative damage, and cell death in the hyperglycemic animal model [71]. In the normal condition, TXNIP is located in the nucleus. Upon oxidative stress, however, TXNIP translocates to cytosol and mitochondria and binds to TRX1 and TRX2, respectively. TXNIP facilitates NLRP3 inflammasome activation either by binding to NLRP3 or translocation to mitochondria and aggravates mitochondrial dysfunction. In the latter, the release of DAMPS to cytosol and recognition by NLRP3 is supposed to stimulate inflammatory response [72]. It has been reported that NLRP3 and TXNIP were activated in patients with degenerate disc [73]. TXNIP/NLRP3 activation led to increased apoptosis in NP cells, indicating the detrimental effect of the inflammatory pathway on IVD cell homeostasis [73]. In another study, NLRP3 inflammasome activation was seen in patients with LBP and the grade of cartilage endplate (CEP) degeneration was correlated with the level of inflammasome complex expression [74]. It was demonstrated that oxidative stress elevated mtROS content, MDA level, and activated TXNIP/NLRP3 signaling pathway as well [74, 75].

## Small molecules targeting mitochondrial dysfunction in IVD degeneration

### Phenolic compounds

#### Curcumin

Curcumin extracted from *Curcuma longa* exerts anti-inflammatory and anti-oxidative properties in different pathobiological states [76–78]. Curcumin can inhibit the NF-κB and MAPK pathways and has been shown to reduce the activity of pro-inflammatory cytokines in degenerate IVD [76, 77]. Posing the ROS scavenging property, curcumin protects mitochondrial homeostasis via inhibition of peroxidase formation and prevention of oxidative damage [78]. Curcumin exerts either pro-apoptotic or anti-apoptotic actions, depending on the context of its application and the cell type that is being studied [79, 80]. Curcumin has been suggested to support disc homeostasis and ameliorates its degeneration via protecting mitochondria and autophagic induction [14]. Kang et al. reported curcumin treatment (˂ 25 μM) reduced ROS production, restored mitochondrial ∆Ψ_m_, and increased ATP production in stress-induced NP cells. Upregulation of Bcl-2 expression and downregulation of Bax, cytosolic cytochrome c, and caspase 9 expression were also shown, indicating the anti-apoptotic effect of curcumin on NP cells in a dose-dependent manner [14].

#### Tri-hydroxy stilbenes

Resveratrol (3,5,4’-trihydroxystilbene) is a naturally occurring phytoestrogen and hydroxylated derivative of stilbene [81, 82]. Resveratrol has been shown to reduce the expression of pro-inflammatory cytokines, TNF-α and IL-1β, in a radiculopathy pain model [83]. It also preserved cytoskeletal structure, scavenged ROS, and restored lost ∆Ψ_m_ which resulted in decreased apoptosis [84]. It was shown resveratrol prevented the loss of ∆Ψ_m_, cellular ATP, and apoptosis against oxidative damage, and enhanced matrix biosynthesis in vivo [85]. The anti-degenerative property of resveratrol on NP cells has been attributed to the autophagy-inducing property of resveratrol through PI3K/Akt signaling pathway [86]. Different strategies have been applied to improve the metabolic stability and bioavailability of resveratrol using carriers and resveratrol derivatives [87]. Polydatin (piceid or resveratrol-3-O-d-glucoside) is a glucoside form of resveratrol with the same properties as well as improved resveratrol drawbacks [88, 89]. Polydatin exerts anti-oxidant and mitochondrial protection through sirtuin 1 upregulation and activation of nuclear factor-erythroid 2-related factor 2/anti-oxidant response element (NRF2/ARE) signaling pathway [90]. In vivo studies have demonstrated that polydatin attenuates the progression of IVD degeneration and enhances matrix biosynthesis and cell proliferation via Nrf2 signaling activation [49]. Restored ∆Ψ_m_ and decrease of ROS production and MDA level were also reported in polydatin-treated CEP chondrocytes [91].

### Flavonoids

Naringenin and its glucoside derivative, naringin, belong to the flavanone subclass of flavonoids [92]. Tomatoes and citrus fruits including oranges and grapefruit are rich in naringenin and naringin [93]. These compounds exert anti-apoptotic and mitochondrial protective effects via activation of PI3K/Akt signaling pathway [94]. Nan et al. demonstrated that naringin can prevent mitochondrial damage and energy deprivation in stress-induced NP-derived mesenchymal stem cells which was attributed to PI3K/Akt signaling pathway [94]. Epigallocatechin-3-gallate (EGCG) is a catechin found abundantly in green tea (*Camellia Sinensis*) with negligible cytotoxicity when concentration is less than 50 μM [95]. EGCG protected ∆Ψ_m_ level against oxidative stress and acted as a pro-survival agent in NP cells via activation of PI3K/Akt signaling pathway [95]. Icariin, isolated from *herba epimedii* or horny goat weed, is a bioactive and peroxylated flavonol glycoside compound, which has been studied for the treatment of degenerative diseases of articular cartilage [96]. Icariin’s anti-oxidative and mitochondrial protective effects are attributed to the activation of PI3K/Akt and Nrf2 signaling pathways, resulting in ∆Ψ_m_ restoration, decrease of ROS production, and apoptosis in NP cells [96, 97]. Furthermore, it was supposed a probable cross talk between Nrf2 and PI3K/Akt for attenuating the mitochondrial damage and preserving the cellular redox homeostasis [97]. Nonetheless, because of rapid intestinal metabolism, icariin utilization is hindered by low bioavailability. Thus, controlled release systems have been introduced in recent years to deliver icariin more efficiently [98].

### Other compounds with anti-oxidant activity

#### NAC

N-Acetyl-l-cysteine or N-acetylcysteine (NAC) is a thiol-containing anti-oxidant with anti-inflammatory, mucolytic, and acetaminophen detoxification activities [99, 100]. NAC exerts its anti-oxidant properties directly by scavenging free radicals such as hydroxyl radical through thiol interaction at a high rate. NAC as a precursor of cysteine enhances glutathione activity in the body, the master anti-oxidant in cells, and the anti-oxidant index, glutathione/oxidized glutathione (GSH/GSSG), is upregulated by NAC treatment [100]. The influence of NAC on cell senescence and apoptosis is controversial, depending on the cell type and the applied experimental conditions [101, 102]. NAC also counteracts mitochondrial dysfunction via enhancing ETC activity and ATP production [17]. The chondroprotective effects of NAC, as determined by the restoration of ∆Ψ_m_, increase the GSH/GSSG ratio, which are lost in a harsh microenvironment characterized by hypoxia, low pH, and high levels of pro-inflammatory inducers [103]. NAC demonstrated cell proliferative and anti-senescent properties in stress-induced NP cells via regulating P16 and P53 expression [104, 105]. Though oral administration of NAC for evaluations in mouse models with degenerate IVD has been exploited, its oral bioavailability is low and is mostly eliminated from the body [106]. Therefore, utilization of vehicles for controlled release and stabilization of NAC has been suggested.

#### Melatonin

Melatonin is released from the pineal gland and regulates circadian rhythm [107]. Enzymatic production of melatonin concisely includes acetylation of serotonin in the presence of serotonin N-acetyl transferase and formation of melatonin via methylation of acetylated serotonin in the presence of hydroxindole-o-methyl transferase [108]. Melatonin deficiency is implicated in the mitochondrial dysfunction and aging [109, 110]. Because of its amphiphilic nature, melatonin can easily penetrate the plasma membrane and reach various subcellular organelles including the nucleus and mitochondria [111]. The proliferative, anti-apoptotic properties and autophagic induction of melatonin in AF cells have also been reported. Melatonin may act as a sirtuin 1 agonist and inhibits apoptosis and calcification in CEP chondrocytes [112]. Findings indicate that there is a correlation between aging, IVD degeneration, and reduction of melatonin secretion. It is hypothesized that melatonin retards the process of aging through its anti-oxidative and free radical scavenging properties as well as prevention of oxidative damage accumulation in cells [113]. Melatonin increased the activity of complexes I and IV of ETC and helped mitochondrial act properly to ameliorate oxidative stress in IVD cells via restoring mitochondrial ∆Ψ_m_, ATP production enhancement, reducing cytochrome c leakage to the cytosol, and apoptosis subsequently [111, 114, 115]. He et al. demonstrated melatonin mitigated the apoptosis in stress-induced NP cells through restoring the lost ∆Ψ_m_ and inhibition of cytochrome c release from mitochondria [114]. Chen et al. obtained similar outcomes after melatonin treatment of oxidative-damaged cells. The level of ∆Ψ_m_ is reduced after tert-butyl hydroperoxide (TBHP) treatment; however, melatonin protected the mitochondrial function via upregulation of Bcl-2 and downregulation of Bax, cytosolic cytochrome c, and caspase 3 expression [115]. Melatonin inhibited mitochondrial-induced apoptosis, improved mitochondrial membrane potential, and enhanced the ECM synthesis in TBHP-treated NP cells. The mitochondrial protective effects of melatonin arise from mitophagy induction in a parkin-dependent manner. Conversely, either cyclosporine A (CsA), a mitophagy inhibitor, or parkin knockdown blunts the protective effects of melatonin [115]. Melatonin also showed the ability to prevent NLRP3 inflammasome priming and activation and inhibited IL-1β secretion [116]. In vitro IL-1β stimulation of NP cells increased the expression of NLRP3 and P20 and the level of mtROS and upregulated NF-κB signaling while the expression of SOD 2 decreased. Melatonin treatment, on the other hand, reduced the NP cells’ inflammatory response via disturbing NLRP3/IL-1β-positive loop, downregulation of NF-κB, and enhancing the anti-oxidant activity of mitochondria. In in vivo evaluations on AF-punctured rat models, melatonin treatment demonstrated the upper expression of collagen II and aggrecan and lower level of NLRP3, P20, and IL-1β in comparison to melatonin+ LPS (NLRP3 inflammasome activator) injection, indicating the anti-degenerative and anti-inflammatory response of melatonin against NLRP3 inflammasome priming and activation [116].

#### Alpha-lipoic acid

ALA is a mitochondrial compound which acts as a co-factor in enzymatic systems including pyruvate dehydrogenase and α-ketoglutarate dehydrogenase complexes in the Krebs cycle [117]. ALA is a thiol-containing anti-oxidant which scavenges ROS; elevates the level of other endogenous anti-oxidants including glutathione, vitamin C, and vitamin E; and reduces lipid peroxidation as well [117]. It acted as an anti-apoptotic agent via increasing mitochondrial ∆Ψ_m_ in high-glucose-induced mitochondrial damage in EPC cells [118].

### Mitochondria-targeted anti-oxidants

**Fig. 2:**
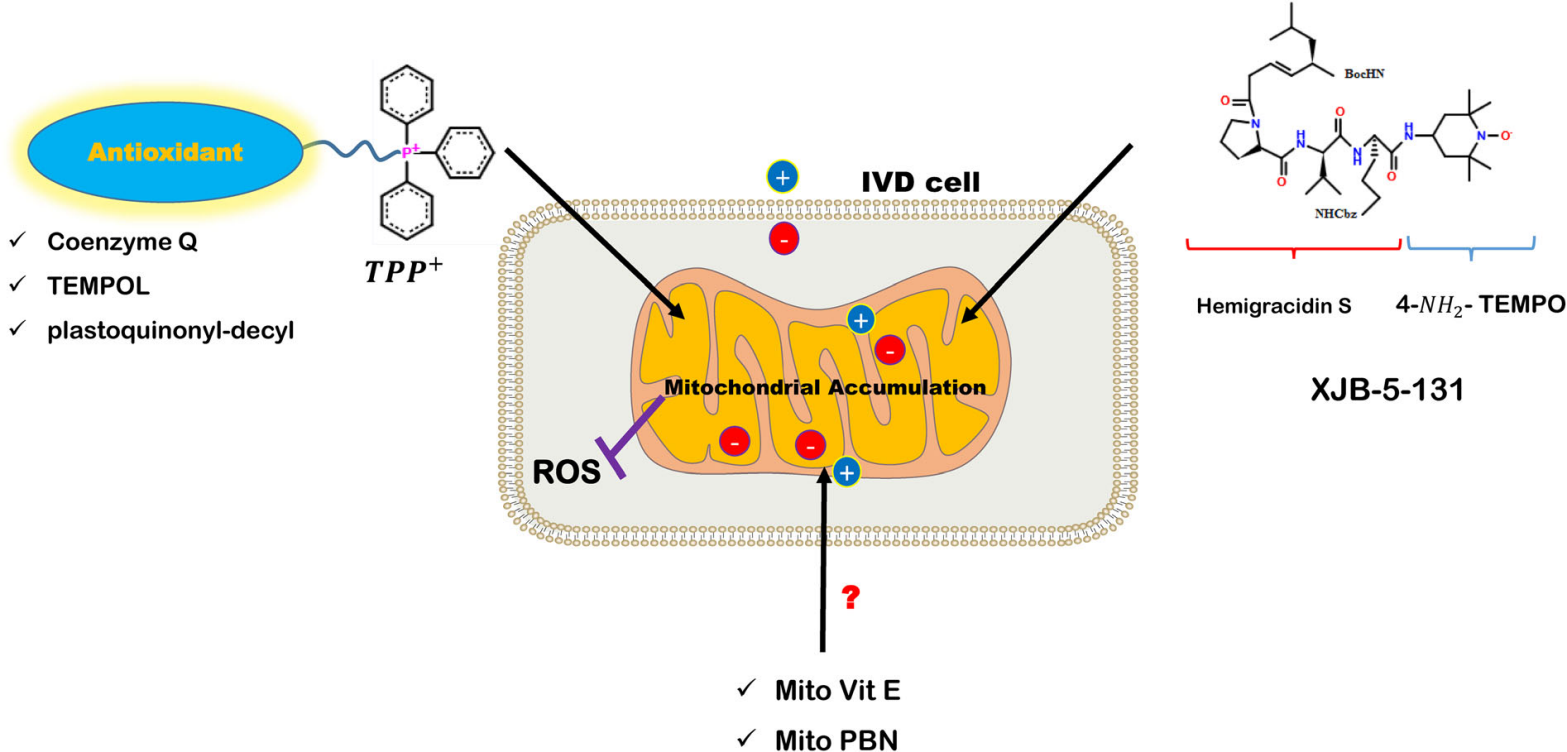
Mitochondria-targeted anti-oxidants using *TPP*^+^ and hemigracidin S as moieties highly accumulate in mitochondria relative to unmodified anti-oxidants

Many anti-oxidants may fail to permeate the cytoplasmic membrane to reach the cytosolic space and target damaged mitochondria. Also, high concentrations of most drugs cause cytotoxicity and cell death. To circumvent these limitations, novel and more objective delivery systems have been introduced for the precise targeting of mitochondria. Mitochondria-targeted moiety anti-oxidant conjugates which are referred to such systems are able to permeate the phospholipid bilayer of cytoplasm and accumulate in mitochondria [119]. Various strategies have been employed to target mitochondrion. In the tetraphenylphosphonium (TPP^+^)-linked ROS scavenger system, TPP^+^ penetrates the negatively charged lipophilic inner-membrane of the cell as a lipophilic and cation motif and delivers the anti-oxidant to mitochondria [119]. Mitoquinone (MitoQ), mitochondrial vitamin E (MitoVitE), (2-(2,2,6,6-tetramethylpiperidin-1-oxyl-4-ylamino)-2-oxoethyl)triphenylphosphonium chloride (MitoTEMPO), mitochondrial alpha-phenyl-tert-butyl-nitrone (Mito PBN), Visomitin (also known as SkQs), and XJB-5-131 are all the compounds with a high potential to attack to the plasma membrane and accumulate in mitochondria multiple folds more than their unmodified counterparts [119, 120] (Fig. 2). Here, we review the mitochondria-targeted anti-oxidants, employed specifically for IVD application.

#### MitoQ

Ubiquinone or coenzyme Q, located in the inner mitochondrial membrane (IMM), is an endogenous anti-oxidant, which transports electrons from complexes I and II to complex IV. Along with CsA, MitoQ also inhibits mitochondrial membrane permeability transition pores (MPTP) opening and subsequent apoptosis in cells which is discussed in the “Other mitophagy- regulatory molecules” section. Previous studies have evaluated the mitochondrial protective effects of CoQ and its exogenous analogs; however, utilization of CoQ analogs is hindered by poor water solubility and auto-oxidation. Idebenone as a CoQ derivative could complement respiration in fibroblasts, while mitochondria-targeted CoQ derivative (MitoQ or ubiquinol) demonstrated to be 800-fold more potent than idebenone to prevent cell death [121]. Studying on compression-induced NP cells, MitoQ restored mitochondrial function and prevented the loss of mitochondrial ∆Ψ_m_ and mitochondria-mediated apoptosis [122]. MitoQ balanced mitochondrial dynamics via upregulation of mitochondrial fusion (Mfn) molecules Mfn1and Mfn2 as well as downregulation of fission molecule Drp1 to prevent cell death. It also activated Nrf2 and its downstream molecules SOD 2 and NQO-1. Ex vivo study on rat tail disc has convincingly demonstrated the in vitro outcomes that MitoQ could re-organize AF collagen fibers, restored the size of NP tissue, and alleviated disc degeneration [122]. Overall, MitoQ would be a potent mitochondrial specific anti-oxidant that preserves mitochondrial homeostasis in NP cells.

#### MitoTEMPO

Piperidine nitroxide radical or TEMPO (2,2,6,6-tetramethyl-1-piperidinyloxy) and its derivatives are anti-oxidants with reversible redox behavior that act as SOD mimetic and ${\mathrm{O}}_2^{.-}$ scavenger [123, 124]. To enhance the selective targeting to mitochondria, MitoTEMPO has been introduced that accumulates in mitochondria several hundredfolds more than untargeted TEMPOs [125]. Song et al. showed that AGE treatment activated NF-κB and NLRP3 inflammasome, promoted pro IL-1β expression, and enhanced mtROS production and mitochondrial membrane permeability in NP cells, while MitoTEMPO treatment alleviated inflammatory response and mitochondrial damage [126]. They reported that MitoTEMPO reduced apoptosis via rescuing mitochondrial ∆*Ψ*_*m*_ and inhibition of cytochrome c release. They claimed sirtuin 3 activation is implicated in the neutralization of AGE treatment. Nonetheless, they did not discuss about a probable MitoTEMPO contribution in activation of sirtuin 3.

#### SkQs

Plastoquinone is implicated in photosynthesis of plant cells as an electron carrier from photosystem II to cytochrome *b*_6_f of IMM [127]. Plastoquinone derivatives, SkQs (“Sk” from acclaimed Russian scholar, Skulachev and “Q” from quinone), are another class of TPP^+^-linked anti-oxidants [128]. SkQ1 (plastoquinonyl-decyl-triphenylphosphonium) is an outstanding SkQ compound, commercially available under the trade name “Visomitin” as eye drops [129]. In comparison to MitoQ, SkQ1 is more efficient to prevent lipid peroxidation, superoxide, and hydroxyl ion formation. SkQ1 has a higher transfer rate than MitoQ to cross the plasma membrane. Anti-oxidant properties of SkQ1 are attributed to (1) uncoupling cycling between cationic moiety (C_12_TPP) of SkQ1 and anionic fatty acid, resulting in reduced ROS level, (2) SkQ1 reaction with lipoperoxyls and reducing to SkQH_2_, and (3) inhibition of superoxide formation at the site I_f_ of complex I and site I_Q_ of complex III and reducing to SkQH_2_ in a recycling way in which SkQ1 is regenerated again [130, 131]. In the AGE-treated NP cells, SkQ1 demonstrated an anti-apoptotic effect that partially reversed Bcl-2 level even more than MitoTEMPO [54]. It also restored mitochondrial ∆*Ψ*_*m*_ and reduced ROS level.

#### XJB-5-131

XJB-5-131 is a hemigracidin-(4-*NH*_2_-TEMPO) conjugate that functions as a mitochondria-targeted anti-oxidant. Hemigracidin as the cell-permeable moiety delivers 4-*NH*_2_-TEMPO to mitochondria [132]. XJB-5-131 prevents ferroptotic cell death and attenuated oxidative damage in aging HdhQ (150) Huntington’s disease mouse model as well [133, 134]. In the aging mouse model, ERCC1^−/∆^, XJB-5-131 improved matrix content in the NP section and cellularity in the CEP as well [59].

A list of small molecules targeting mitochondrial dysfunction and their biological effects are brought in Table 1.

**Table 1:** Small molecules targeting mitochondrial dysfunction in IVD cells and their biological consequences

Compound (molecular weight, g/mol)	Conditioning method	Biological consequences	References
Curcumin (368.38)	TBHP-treated NP cells	∆*Ψ*_*m*_ preservation, increased ATP production, anti-apoptosis in a dose-dependent way (concentration < 25 μM)	[14]
Resveratrol (228.25)	Sodium nitroprusside-induced NP cells	Decreased intracellular ROS, ∆*Ψ*_*m*_ preservation, anti-apoptosis	[84]
	*H*_2_*O*_2_-treated NP cells	∆*Ψ*_*m*_ preservation via PI3K/Akt signaling pathway	[85]
	*H*_2_*O*_2_-treated NP cells	Autophagy induction via PI3K/Akt signaling pathway, anti-degenerative	[86]
Polydatin (390.4)	TNF-α-treated NP cells, AF-punctured rat model	Inhibited cell senescence, activated Nrf2 both in vitro and in vivo, preserved ECM homeostasis, and alleviated IVD degeneration	[49]
	H_2_O_2_-treated EPC	∆*Ψ*_*m*_ preservation, decrease of mtROS, parkin activation, and Nrf2 upregulation	[91]
Naringin (580.5)	H_2_O_2_-treated NPMSCs	∆*Ψ*_*m*_ preservation, enhanced ATP level, decreased ROS content, anti-apoptosis (concentration < 100 μM)	[94]
Icariin (676.7)	H_2_O_2_-treated NP cells AF-punctured rat model	Activated Nrf2 and upregulated Nrf1 and TFAM, inhibited cytochrome C release from mitochondria, and apoptosis ameliorated IVD degeneration via Nrf2 upregulation	[97]
EGCC (458.4)	H_2_O_2_-treated IVD cells	∆*Ψ*_*m*_ preservation, anti-apoptosis via PI3K/Akt signaling pathway (concentration < 50 μM)	[95]
NAC (163.2)	Mechanical-loaded NP cells	Downregulated P53 and 16 expressions and inhibited senescence	[104]
	Hyper-osmolality-treated NP cells	Decreased intracellular ROS, and inhibited senescence	[105]
Melatonin (232.28)	TBHP-treated EPC, AF-punctured rat model	Sirtuin 1 activation, inhibited EPC calcification	[112]
	H_2_O_2_-treated NP cells	Inhibited mitochondrial injury by ∆*Ψ*_*m*_ preservation, inhibited mitochondria-mediated apoptosis	[114]
	TBHP-treated NP cells, AF-punctured rat model	Anti-apoptotic, preserved ECM homeostasis via mitophagy induction both in vitro and in vivo	[115]
	IL-1β-treated NP cells	Attenuated NLRP3 inflammasome activation, decreased mtROS level	[116]
ALA (206.3)	High-glucose-treated EPC	Decreased ROS level, inhibited mitochondria-mediated apoptosis, ∆*Ψ*_*m*_ preservation	[118]
MitoQ (678.8)	Compression-loaded NP cells	Activated Nrf2 and upregulated downstream genes, SOD2 and NQO-1, regulated mitochondrial dynamic molecules, upregulation of Mfn1 and 2, and downregulation of Drp1, ∆*Ψ*_*m*_ preservation	[122]
MitoTEMPO (511)	AGE-treated NP cells	Alleviated mitochondrial membrane transition pore opening, decreased mtROS level, anti-apoptosis, ∆*Ψ*_*m*_ preservation	[126]
SkQ1 (617.6)	AGE-treated NP cells	Alleviated mitochondrial membrane transition pore opening, decreased mtROS level, anti-apoptosis	[54]
XJB-5-131 (959.2)	Accelerated aging ERCC1^−/∆^ mouse model	Ameliorated IVD degeneration, enhanced disc ECM content	[59]

## Mitophagy and its regulatory small molecules

Mitophagy (mitochondrial autophagy) is a type of selective autophagy occurring as a response to mitochondrial dysfunction which results from excessive ROS accumulation and/or loss of ∆Ψ_m_.

Mitophagy is a homeostatic cellular process to remove excessive or dysfunctional mitochondria and preserve cellular energy metabolism. Pathological stimuli including oxidative stress as well as aging lead to impaired mitophagy in IVD. Impaired mitophagy leads to the loss of cellularity and apoptosis. The correlation between apoptosis and mitophagy has been extensively researched. Zhang et al. showed that parkin expression and mitophagy are upregulated as the IVD degenerates, and this upregulation has a direct relationship with the stage of degeneration. They reported that impaired mitophagy with accumulated P62 exacerbates apoptosis in NP cells, while salidroside improved autophagosome-lysosome fusion which consequently abated apoptosis and mitigated disc degeneration [135]. Conversely, Xu et al. reported that TBHP+ FCCP treatment resulted in more apoptosis relatively to only TBHP-treated NP cells, indicating the role of excessive mitophagy in apoptosis induction [136]. They argued that multiple factors including exposure time and concentration of stressors as well as the degree of degeneration determine the beneficial or detrimental role of mitophagy to suppress IVD degeneration. So far, the optimal level of mitophagy needed for IVD regeneration has not been determined. Nonetheless, it is obvious that oxidative stress induces mitophagy in the IVD, which is intensified with the degree of degeneration. Also, a regulated level of mitophagy might be useful through the elimination of depolarized mitochondria.

**Fig. 3:**
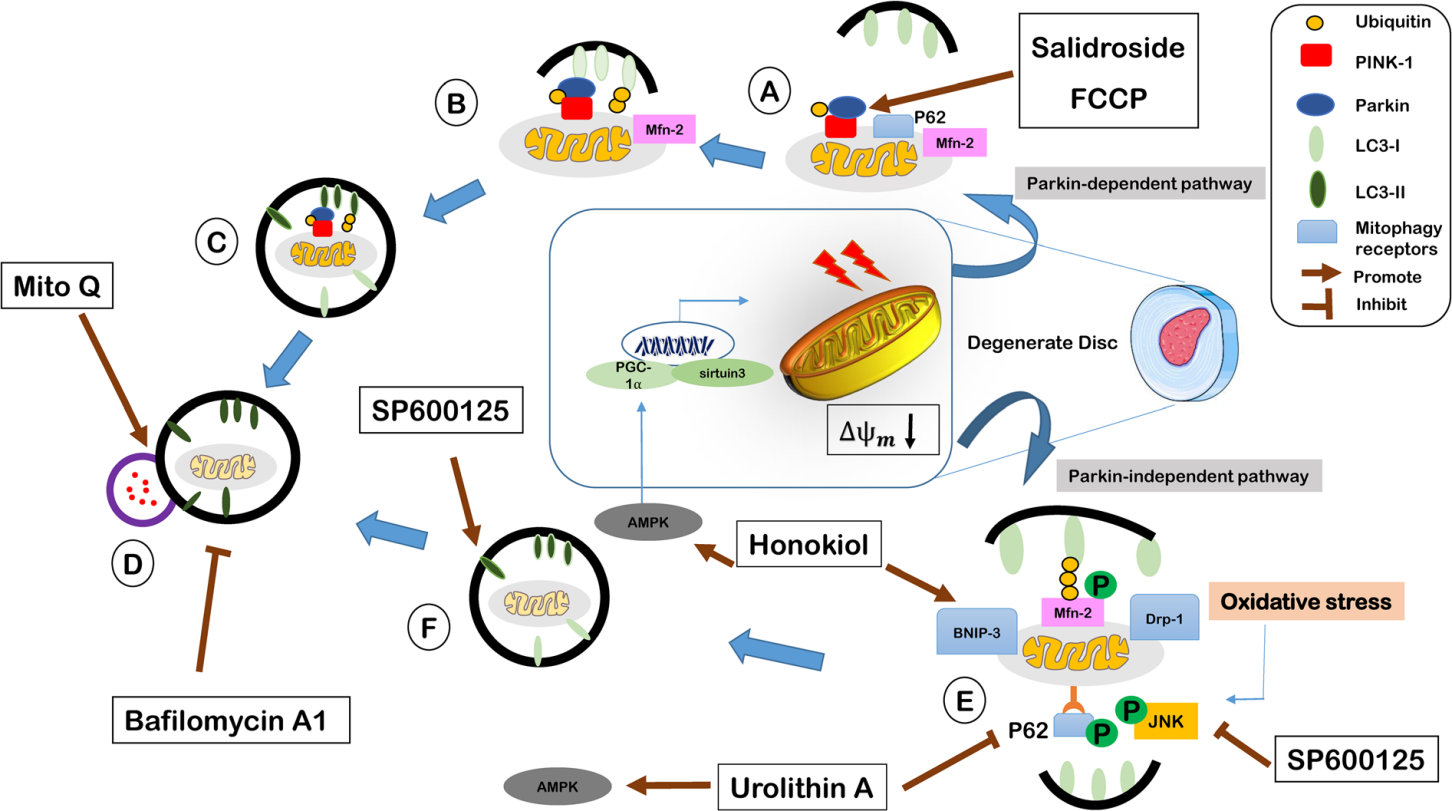
Mitophagy pathways and regulatory small molecules for IVD cells: mitophagy in IVD cells takes place via two pathways: (i) Parkin-dependent: salidroside and FCCP as mitophagy activators enhance parkin level. Salidroside also downregulates P62 expression. Mito Q facilitates P62 degradation and leads to lysosomal digestion. (ii) Parkin independent: SP600125 (JNK inhibitor) inhibits JNK phosphorylation and LC3-II accumulation in oxidative stress-induced bone marrow-derived mesenchymal stem cells. Honokiol upregulates mitochondria dynamic molecules, Mfn-2 and Drp-1, expression, and activates the mitophagy receptor, BNIP3. Bafilomycin A1 inhibits lysosomal degradation of autophagosome and LC3-II accumulation. **A** PINK-1 phosphorylation and parkin ubiquitination, **B**, **E** mitophagy receptors activation, **C**, **F** autophagosome formation, **D** lysosomal digestion

Regulated mitophagy can enhance cellular survival and delay aging by inducing cellular proliferation and inhibiting cellular senescence. In contrast, both excessive and insufficient mitophagy result in energy deprivation, ROS accumulation, and oxidative stress, which eventually lead to cell death. Understanding how mitophagy regulates IVD homeostasis, and targeting influential molecules and signaling pathways, has been considered in recent years. In mitophagy, the damaged mitochondrion is engulfed in the autophagosome and is degraded by lysosome [137]. This process takes place either in the canonical (parkin dependent) pathway or in the non-canonical (parkin independent) pathway (Fig. 3). Initiation of each pathway depends on the activation of various upstream molecules and signals which are described later.

### The canonical pathway

In the canonical pathway, PINK-1 recruits parkin from the cytoplasm to OMM. Upon PINK-1 phosphorylation and parkin ubiquitination, mitophagy receptors such as P62 are activated, and mitophagosome is formed via interaction of mitophagy receptors and autophagy protein, LC3. Autophagosome is fused with the lysosome and is degraded [137]. Regulation of mitophagy is conducted through controlling the expression of parkin, PINK-1, and mitochondrial dynamics molecules [138].

PINK-1 silencing enhances NP cell senescence and ROS production either in a normal or oxidative stress-induced state, showing how mitophagy controls cell proliferation, aging, and perhaps IVD degeneration. Kang et al. observed that the impaired mitophagy in the compression-induced human NP cells was attributed to the decreased activity of lysosomal enzymes [122]. Parkin knockdown promotes apoptosis and mitochondrial impairment in NP cells [135]. Nevertheless, salidroside, a glucoside of tyrosol which is extracted from rhodiola, improves mitophagy through upregulation of parkin, inhibition of cytoplasmic accumulation of P62, and increasing the LC3-I to LC3-II conversion that is necessary for mitophagy initiation. Salidroside suppressed apoptosis by repairing the impaired mitochondria [135].

### Non-canonical pathway

Mitophagy may be initiated through the parkin-independent pathway, non-canonical pathway, and occurs via various pathways including BNIP3/NIX, JNK/MAPK, HIF-1α, and PI3K/Akt/mTOR. Upon mitochondrial damage, various autophagy receptors such as FUNDC1, NIX, and BNIP3 are recruited in the outer mitochondrial membrane. Autophagosome is formed through the interaction of autophagy receptors and LC3 protein that finally will be removed via lysosomal destruction [137]. It has been found that honokiol upregulates Drp-1 and Mfn-2 and increases mitophagy in the sirtuin 3-dependent manner which was determined by upregulation of BNIP3 and the ratio of LC3-II to LC3-I [139].

Autophagy and mitophagy might be also activated in a JNK-dependent manner [140, 141]. JNK/MAPK pathway is initiated as a response to inflammation signals or oxidative stress [140, 142]. Wang et al. reported a non-canonical and JNK-dependent mitophagy activation in bone marrow-derived mesenchymal stem cells, which is a common cell source for cartilage and IVD regeneration [141]. They found that oxidative stress induction for a limited period of time (< 1 h) activates mitophagy in a JNK-dependent manner. To corroborate their findings, both mitophagy inhibitor (CsA) and JNK inhibitor (SP600125) increased apoptosis and catabolic activity. However, antimycin A constricted apoptotic cell death through mitophagy activation [141]. Urolithins are microbiota-derived gut metabolites, which are converted from ellagitannins available in pomegranate and some types of berries with anti-senescent and anti-degenerative properties in NP cells and IVD, respectively [143–145]. In particular, urolithin A has shown to be a potent mitophagy activator, being able to preserve IVD cell homeostasis [146]. In NP cells, urolithin A suppressed intrinsic apoptosis pathway and relatively restored ∆*Ψ*_*m*_ level which was lost by TBHP treatment. It was argued that urolithin A activates mitophagy in NP cells via the AMPK signaling pathway activation [146].

### Other mitophagy regulatory molecules

- Mitochondrial membrane permeability transition pore inhibitory molecules

Identification of molecules that are capable of inhibiting the opening of MPTP is another way to regulate mitophagy in IVD cells [147]. These molecules include CsA and CoQ that inhibit mitochondrial permeability and electron leakage to the cytosol. Adenine nucleotide translocate (ANT) that translocates AT/ADP across the mitochondrial membrane binds with CyP-D protein, and opens the MPTPs; however, the formation of this complex is inhibited in the presence of CsA [147]. CsA as a common mitophagy inhibitor has been widely exploited in IVD cells [115, 138, 141, 148].

The CoQ family is lipid-soluble small molecules with anti-oxidant and anti-inflammatory properties [149, 150]. MitoQ as a form of CoQ has been used for the repair of impaired mitophagy in the compression-induced human NP cells that was attributed to the decreased activity of lysosomal enzymes [122]. However, utilization of MitoQ counteracted the defective mitophagy flux by restoring the acidity of lysosome and consequently enhancement of P62 degradation. MitoQ also double activated the mitophagy via the PINK-1/Park signaling pathway.

- Mfn-2

Mfn-2, a protein located in the mitochondrial outer membrane, is an indispensable factor for mitochondrial fusion. With increase of mitochondrial membrane permeability and parkin recruitment, Mfn-2 is ubiquitinated. Mfn-2 ubiquitination is associated with the activation of mitophagy and elimination of dysfunctional mitochondria. Mfn-2 acts in a PINK-1/parkin-dependent manner and activates mitophagy [151, 152]. Expression of Mfn-2 decreases as the degree of NP degeneration increases. Mfn-2 exerts an anti-apoptotic effect via ROS-dependent mitophagy and alleviates IVD degeneration. Recent findings indicate that Mfn-2 knockdown enhances apoptosis in NP cells and blocks autophagy, while Mfn-2 overexpression mitigates apoptosis through PINK1/parkin pathway and in a ROS-dependent manner [138].

- NDUFA4L2

NDUFA4L2 is a mitochondrial gene, located in the complex I of ETC, and is implicated in the electron transport and mitochondrial respiratory system. NDUFA4L2 is activated in the response of HIF-1α upregulation, a crucial factor for cell proliferation and homeostasis of ECM in the hypoxic microenvironment of IVD [136, 153]. Under hypoxic conditions, HIF-1α signaling is activated and upregulates NDUFA4L2 expression, resulting in inhibition of complex I activity, reduction of ROS production, and enhancement of cellular survival [154, 155]. Studies on NP cells suggest that the anti-apoptotic effect of HIF-1α/NDUFA4L2 may be attributed to its mitophagy regulatory behavior [136]. In vivo studies by Xu et al. demonstrated that the expression levels of NDUFA4L2 and HIF-1α decrease as the progression of IVD degeneration increases. Conversely, injection of adeno-NDUFA4L2 lowered the levels of caspase 3 and parkin [136]. In addition, the mitophagy activator carbonyl cyanide 4-(trifluoromethoxy) FCCP led to excessive mitophagy and induced apoptosis [136]. In vitro evaluation of NP cells also determined that mitochondrial protective effect of NDUFA4L2 occurs via activation of HIF-1α signaling pathway [136]. Therefore, targeting NDUFA4L2 may be a promising approach to protect mitochondrial function and enhance NP cell survival.

- Mitochondrial biogenic molecule PGC-1α

Peroxisome proliferator-activated receptor (PPAR)-ɣ co-activator 1α (PGC-1α) acts as a co-factor for thermogenesis and mitochondrial bioenergetics in cells. It is the master regulator of mitochondrial bioenergetics and preserves redox homeostasis via ROS detoxification. Findings in IVD cells have demonstrated that oxidative stress-induced mitophagy aggravates apoptosis and accelerates cellular senescence [54, 148]. Conversely, PGC-1α activation downregulates mitophagy and acts as an anti-apoptotic factor. Xu et al. found that PGC-1α overexpression mitigated the apoptosis upregulation via controlling the excessive mitophagy in a sirtuin 2-dependent manner. Song et al. also reported that PGC-1α alleviates apoptosis and mitophagy through the AMPK/PGC-1α/sirtuin 3 signaling pathway [54].

The list of small molecules employed to regulate mitophagy and their mechanism of action are brought in Table 2.

**Table 2:** Mitophagy activators and their mechanism of action in IVD cells

Small molecule (molecular weight, g/mol)	Concentration	Therapeutic target (molecule/signaling pathway)	Therapeutic outcome	Reference
MitoQ (678.8)	200–500 nM	PINK1/parkin activation	Repair of impaired mitophagy (P62↓)	[122]
Honokiol (266.3)	< 10 μM	AMPK/PGC-1α/sirtuin 3 activation	Anti-oxidant activity (SOD)↑	[139]
			Cell senescence↓	
Salidroside (300.3)	200 μM	Parkin activation	Repair of impaired mitophagy (P62↓)	[135]
Urolithin A (228.2)	0–20 μM	AMPK activation	Apoptosis↓, ∆*Ψ*_*m*_ restoration, regenerated degenerate disc	[146]

## Summary

In this review, we have discussed the role of mitochondrial dysfunction in IVD degeneration and explored the interplay with oxidative stress and inflammation. Consequences of oxidative stress such as apoptosis, cell senescence, and the production of MDA, AOPP, and AGEs are implicated in IVD cell mitochondrial dysfunction. ROS levels in mitochondria are associated with the inflammatory pathway and the NLRP-3 inflammasome. Targeting mitochondrial dysfunction with compounds such as NAC, melatonin, ALA, and phenolic and flavonoid compounds results in lowered ROS content, preserves ∆Ψ_m_, and promotes ECM homeostasis in IVD cells. In addition, mitochondria permeable small molecules such as *TPP*^+^-linked anti-oxidants and XJB-5-131 have been shown to be efficient for mitigating mitochondrial dysfunction and alleviating IVD degeneration. Most of the in vivo studies done to date have concentrated on rodent models. However, the objective evaluation of these drugs should be carried out in more appropriate translational models that are closer to the human in vivo situation, including animal models that resemble the developmental aspects of the human IVD, particularly the non-persistence of notochordal cells. Molecules regulating mitophagy were also discussed, and it was concluded that insufficient mitophagy induces apoptosis and leads to IVD degeneration. It is also unclear whether small molecules are effective in humans. In conclusion, future clinical trials should focus on studying the safety and efficacy of compounds that promote mitophagy and alleviate mitochondrial dysfunction for treating IVD degeneration and preserving the structure and function of the IVD.

## Abbreviations

AF: Annulus fibrosis
AGEs: Advanced glycation end products
ALA: Alpha-lipoic acid
AOPP: Advanced oxidation protein products
ARE: Anti-oxidant response element
ASC: Apoptosis-associated speck-like protein, containing a caspase-recruitment domain
ATG: Autophagy protein
ATP: Adenosine triphosphate
Bcl-2: B cell lymphoma
bFGF: Basic fibroblast growth factor
BMPs: Bone morphogenetic proteins
CEP: Cartilage endplate
CML: Carboxymethyllysine
CsA: Cyclosporine A
DAMPs: Damage-associated molecular patterns
(∆*Ψ*_*m*_): Mitochondrial membrane potential
ECM: Extracellular matrix
EGCG: Epigallocatechin-3-gallate
ETC: Electron transport chain
FCCP: Carbonyl cyanide 4-(trifluoromethoxy) phenylhydrazone
GSH: Glutathione
GSSG: Oxidized glutathione
GSH/GSSG: Glutathione/oxidized glutathione ratio
GSk-3: Glycogen synthase kinase 3
HIF-1α: Hypoxia inducible factor 1-α
IL-1β: Interlukin-1beta
IVD: Intervertebral disc
Keap1: Kelch-like ECH-associated protein 1
LBP: Low back pain
LPS: Lipopolysaccharides
MAPK: Mitogen-activated protein kinase
Mito PBN: Mitochondrial alpha-phenyl-tert-butyl-nitrone
MitoQ: Mitoquinone
MitoTEMPO: 2,2,6,6-Tetramethyl-4-[[2-(triphenylphosphonio)acetyl]amino]-1-piperidinyloxy,monochloride
MitoVitE: Mitochondrial vitamin E
MDA: Malondialdehyde
MRC: Mitochondrial respiratory chain
mtDNA: Mitochondrial DNA
mtROS: Mitochondrial ROS
NAC: N-Acetyl-l-cysteine or N-acetylcysteine
NF-κB: Nuclear factor kappa B
NLRP: Nucleotide-binding domain, leucine-rich repeat protein
NP: Nucleus pulposus
Nrf2: Nuclear factor-erythroid 2 related factor 2 (Nrf2)
OMM: Outer mitochondrial membrane
OXPHOS: Oxidative phosphorylation
PAMPs: Pathogen-associated molecular patterns
RAGE: Toll-like receptor (TLR)-receptor for AGE
RNS: Reactive nitrogen species
ROS: Reactive oxygen species
RMDs: Rheumatic and musculoskeletal diseases
SOD: Superoxide dismutase
TBHP: Tert-butyl hydroperoxide
TEMPO: 2,2,6,6-Tetramethyl-1-piperidinyloxy
TGF-β1: Transforming growth factor-β1
TNF-α: Tumor necrosis factor-alpha
TRX-1: Thioredoxin 1
TRX-2: Thioredoxin 2
TXNIP: Thioredoxin-interacting protein
